# Associations Between Independent Assessments of Child Appetite Self-Regulation: A Narrative Review

**DOI:** 10.3389/fnut.2021.810912

**Published:** 2022-01-27

**Authors:** Maria A. Papaioannou, Nilda Micheli, Thomas G. Power, Jennifer O. Fisher, Sheryl O. Hughes

**Affiliations:** ^1^Department of Pediatrics, United States Department of Agriculture/Agricultural Research Service (USDA/ARS) Children's Nutrition Research Center, Baylor College of Medicine, Houston, TX, United States; ^2^Department of Human Development, Washington State University, Pullman, WA, United States; ^3^Department of Social and Behavioral Sciences, Center for Obesity Research and Education, Temple University, Philadelphia, PA, United States

**Keywords:** appetite self-regulation, children, methodology, observational tasks, questionnaires, self-report

## Abstract

A variety of eating behaviors among children have been associated with obesity risk and are thought to broadly reflect child appetite self-regulation (ASR). While ASR is thought to occur on cognitive, emotional, motivational, biological, and behavioral levels, the inter-relatedness of ASR constructs as assessed by different methods/measures is not well-characterized. This narrative review describes the correspondence between different methods/measures of child ASR constructs as assessed by self-report questionnaires and/or observational tasks and their relationship to child standardized body mass index (BMIz). Research involving at least two different methods/measures is presented including observational tasks such as the Eating in the Absence of Hunger task, compensation trials, and eating rate, as well as various child eating behavior self-report questionnaires. Keyword searches in the PubMed and PsycINFO databases for articles published between 2000 and July 2021 identified 21,042 articles. Eighteen articles met the inclusion criteria and examined at least two of the targeted measures. Studies comparing questionnaire data with other questionnaire data showed the most evidence of significant associations (*r* values ranging from −0.45 to 0.49), whereas studies comparing questionnaires with observational tasks mostly showed weak (*r* values ranging from −0.17 to 0.19) or not significant associations, with only few studies finding moderate associations (*r* values ranging from −0.38 to 0.33). Studies comparing different observational tasks showed no significant associations. Overall, studies comparing self-report questionnaires showed the most correspondence, whereas those comparing observational tasks showed no correspondence. Studies across methods (questionnaires with tasks) showed less correspondence. Significant associations were found between ASR constructs and child BMIz across five studies using self-report questionnaires and two studies using observational tasks. Future research is needed to clearly define the various ASR constructs, their expected correspondence, and the strength of that correspondence, as well as the relations between ASR constructs and child weight among youth with and without overweight/obesity.

## Introduction

Child appetite self-regulation (ASR) has been identified as a central mechanism in the development of childhood obesity and has been targeted as a modifiable target in childhood obesity prevention programs (1–6). Definitions of ASR span multiple disciplines including the developmental sciences, nutrition, clinical psychology, and public health. Using a biopsychosocial framework, Russell and Russell (7) recently described ASR as multidimensional latent construct that occurs at “…cognitive, emotional, motivational, biological, and behavioral levels” and can be conceptualized in at least three ways. In the first conceptualization, top-down cognitive processes of ASR, such as inhibitory control, are thought to moderate bottom-up biologically drives toward food approach and avoidance. Top-down processes reflect effortful and executive control, whereas bottom-up processes reflect reactive, automatic processes that have neural origins. A second conceptualization included behavioral manifestations of ASR such as food choices and consumption as well as regulatory elements of hunger, satiation (during eating; brings meal to end), and satiety (after end of eating; prevents eating again before hunger). Lastly, ASR can be conceptualized as a process, a trait, or a skill (7), all of which can be measured using different methods. For example, ASR as a process or a skill can be measured using observational methods (e.g., Eating in the Absence of Hunger protocol), whereas ASR as a trait can be measured using a survey (e.g., the Children's Eating Behavior Questionnaire). While these recent theoretical advances provide a robust conceptual framework, measurement of ASR remains quite varied, and the inter-relatedness of different ASR constructs as measured by the various methods and measures is not well-characterized.

The present narrative review was undertaken to evaluate the correspondence of different methods/measures that have been used to assess common ASR constructs that are relevant to obesity risk in young children. Drawing on current multidimensional conceptualizations of ASR (7) and reviews of the extant literature on child obesogenic eating behaviors (8, 9), we focused on commonly used measures of ASR constructs that reflect multiple aspects of ASR (e.g., top-down processes, satiation) but predominantly emphasize reactive bottom-up processes. The current review focused on original research studies that included self-report questionnaires and/or observational tasks to assess common ASR constructs. Observational tasks measuring ASR constructs included compensation trials, Eating in the Absence of Hunger (EAH), and eating microstructure (i.e., eating rate and bite size). ASR self-report measures included the Children's Eating Behavior Questionnaire (parent-report), the Dutch Eating Behavior Questionnaire (parent- and child-report), the Eating in the Absence of Hunger Questionnaire (parent- and child-report), and the children's Self-Regulation in Eating scale (parent-report) by Tan and Holub. A brief description of the measures is given below to illustrate the diversity of measurement approaches and operational definitions employed in the study of ASR among children.

Among observational tasks, compensation trials have been used to assess satiation in children. Specifically, compensation protocols typically characterize the extent to which children adjust food intake at an *ad libitum* meal in response to the energy content of a compulsory preload consumed prior to the meal (10, 11). In other words, this protocol addresses whether children overeat, undereat, or accurately compensate at meals for prior intake. The EAH task assesses satiety by measuring children's intake of palatable foods (i.e., sweet and savory snacks) provided after a meal (along with a stack of toys) (12). Finally, average eating rate and average bite size are used to assess the eating microstructure, often in the context of satiation, by characterizing the number of mouthfuls eaten per minute and by gram, respectively (13, 14). Faster average eating rates and larger average bite sizes are thought to promote excessive intake by outpacing internal satiation signals (13, 14).

Among ASR self-report measures, the most commonly used are the Children's Eating Behavior Questionnaire (CEBQ; parent-report) (15) and the Dutch Eating Behavior Questionnaire [DEBQ; parent- (16) and child-report (17)]. The CEBQ measures eight appetitive traits of children 2 years old and above across 35 items using a 5-point Likert scale. Four of the eight traits are food approaching (i.e., food responsiveness or how responsive a child is to food/eating, emotional overeating, enjoyment of food, and desire to drink) and four are food avoidant (i.e., satiety responsiveness or how responsive a child is to feelings of fullness, emotional undereating, slowness in eating, and food fussiness) (15). The child-report of the DEBQ measures emotional eating, external eating, and restrained eating in children ages 7–12 years across 20 items using a 3-point scale (17). The parent-report of the DEBQ (parent report of child behaviors) measures the same constructs across 30 items on a 5-point scale (16). In addition to the CEBQ and the DEBQ, there are a number of other tools that have been used to assess ASR. For example, the Eating in the Absence of Hunger Questionnaire has two parallel versions, a parent-report of child behaviors (EAH-PC) (18), and a child-report (EAH-C) (19) used with children ages 8–18 years. Both versions assess the frequency of eating in the absence of hunger and specifically measure external eating, negative affect, and fatigue/boredom across 14 items on a 5-point Likert scale. Tan and Holub's children's Self-Regulation in Eating scale (SRES; parent-report) assesses parental beliefs regarding child's ability to self-regulate eating across 8 items on a 5-point Likert scale (20).

Considering the difficulty of operationalizing and explicitly measuring child ASR as well as the various assessment methods available, it is important for research and prevention efforts to understand how ASR constructs as assessed by different methods/measures are related (21). For example, caloric compensation, as measured by compensation trials is thought to be a behavioral analog or manifestation of satiety responsiveness, as measured by the CEBQ (22, 23). While it is not uncommon to employ multiple measures of ASR (24–27), little research to date has been undertaken with the specific goal of characterizing the correspondence of ASR constructs. Further, patterns of associations have been mixed, with some studies utilizing independent measures showing weak associations between ASR measures (25, 26) and others showing no significant associations (24, 27). While ASR is often described in general terms, it is thought to occur at multiple levels and be manifested across a wide range of dimensions. Characterizing the inter-relatedness of ASR constructs as measured by different methods/measures is critical to advance theoretical understanding of the role of ASR in obesity risk and prevention during early childhood.

In this context, the purpose of this narrative review is to describe the correspondence of methods/measures of common ASR constructs relevant to obesity risk in children and to examine the associations between different methods/measures and child standardized body mass index (BMIz). The review focuses on original research studies that included at least two ASR assessment methods (self-report questionnaires and observational tasks) as well as measures within each methodology (i.e., a study including at least two self-report questionnaires or at least two observational tasks). Measures chosen within each methodology were those that are notably related to child obesity risk in the current literature. The review also focuses on children ages 2–12 years for two reasons: (1) eating behaviors mainly develop during this period and (2) this is the time when children are still somewhat dependent on their caregivers while becoming more autonomous and independent in their food choices (28).

## Methods

This narrative review of the literature involved an iterative process of searching for original research articles that included at least two assessments of child ASR constructs from self-report questionnaires and observational tasks. Self-report questionnaires included parent reports of child behaviors as well as child self-reports. We focused on the following constructs that are applicable to ASR: food responsiveness, satiety responsiveness, emotional overeating, external eating, eating in the absence of hunger, eating rate, bite size, slowness in eating, caloric or energy compensation, and satiation and satiety. During this process, additional constructs emerged (e.g., children's self-regulation in eating). Table 1 provides an overview of the constructs, their definitions, and respective assessment tools.

**Table 1:** Conceptualizations and assessment tools of constructs.

**Construct**	**Conceptualization**	**Assessment tool**
Food responsiveness	Responsiveness to external food cues, such as the sight or smell of food, that encourage eating, potentially to excess (8).	CEBQ
Satiety responsiveness	Ceasing consumption in response to internal signals, which may include gut hormone release and gastric distension (8).	CEBQ
Slowness in eating	Slow speed of eating (25).	CEBQ
Emotional overeating	Eating more food during negative emotional states (15).	CEBQ
Emotional eating	Excessive eating in response to emotional states such as anger, fear or anxiety (29).	DEBQ
External eating	Eating in response to food stimuli without regard to internal hunger or satiety (29).	DEBQ
Eating in the absence of hunger	Eating when exposed to palatable (sweet and savory) foods in the absence of hunger (30).	EAH protocol
Eating rate	Energy intake divided by meal duration (25).	Observed
Bite size	Energy intake divided by number of bites (13).	Observed
Caloric/energy compensation	Compensation for energy consumed in a preload during a subsequent *ad libitum* meal (10).	Observed
Satiation	Signals and processes that occur during the course of a meal that bring the meal to an end (31).	N/A
Satiety	Signals and processes that, following the end of a meal, inhibit eating before hunger returns (31).	N/A
Children's self-regulation in eating	Children's regulation of food intake based on internal cues of satiety (32).	SRES

*CEBQ, Children's Eating Behaviors Questionnaire (15); DEBQ, Dutch Eating Behavior Questionnaire (29); EAH, Eating in the absence of hunger (30); SRES, Self-Regulation in Eating Scale (32)*.

### Review Question

The focus of the review was to examine correlational data between different ASR constructs among children as assessed by at least two different methods/measures (self-report questionnaires and observational tasks). We excluded reports of correlations between subscales of the same questionnaire because they do not represent independent assessments.

### Search Strategy

Keyword searches were conducted in electronic databases (PubMed and PsycINFO) in July 2021 using the following terms: (appetitive traits) OR (appetite self-regulation) OR (appetite regulation) OR (child eating behaviors) OR (bite size) OR (eating in the absence of hunger) OR (energy compensation) OR (caloric compensation) OR (food responsiveness) OR (emotional overeating) OR (satiety responsiveness) OR (slowness in eating) OR (emotional eating) OR (external eating) OR (disinhibited eating) OR (satiation) OR (satiety) OR (compensation AND eating). As the focus of the review was on correlations found in the literature, the publication type was limited to original articles, and thus systematic reviews with or without meta-analysis, conceptual articles, case-studies, and dissertations were excluded. We searched for articles published between 2000 and 2021 targeting children 2–12 years old. NM conducted the search in PubMed, which resulted in 20,593 articles. MAP conducted the search in PsycINFO, which resulted in 449 articles. A total of 373 articles in the PubMed search were also found in the PsycINFO search. Relevant articles were also hand-searched to identify any studies that were not included in our search.

### Eligibility Criteria

Studies that met all the following criteria were included in the review: (1) study design (cross-sectional study, longitudinal study, randomized controlled trial), (2) population (children ages 2–12 years and/or their caregivers), (3) articles comparing results of at least two assessments that were originally designed to measure healthy ASR, and (4) article type (peer-reviewed publication). Exclusion criteria included: (1) articles focusing on children with eating disorders (e.g., loss of control of eating, binge eating) and/or developmental disorders that may affect appetite regulation (e.g., autism), (2) articles presenting research that was not original (i.e., review articles, conceptual articles, case-studies, and dissertations), (3) articles measuring child ASR constructs that are not typical/healthy (e.g., disinhibited or restrained eating due to dieting or disordered eating), (4) articles presenting data already presented in a previous publication, (5) articles that measured constructs only by a single item on a questionnaire, and (6) language (title, abstract, and/or full text not in English).

### Study Selection

**Figure 1:**
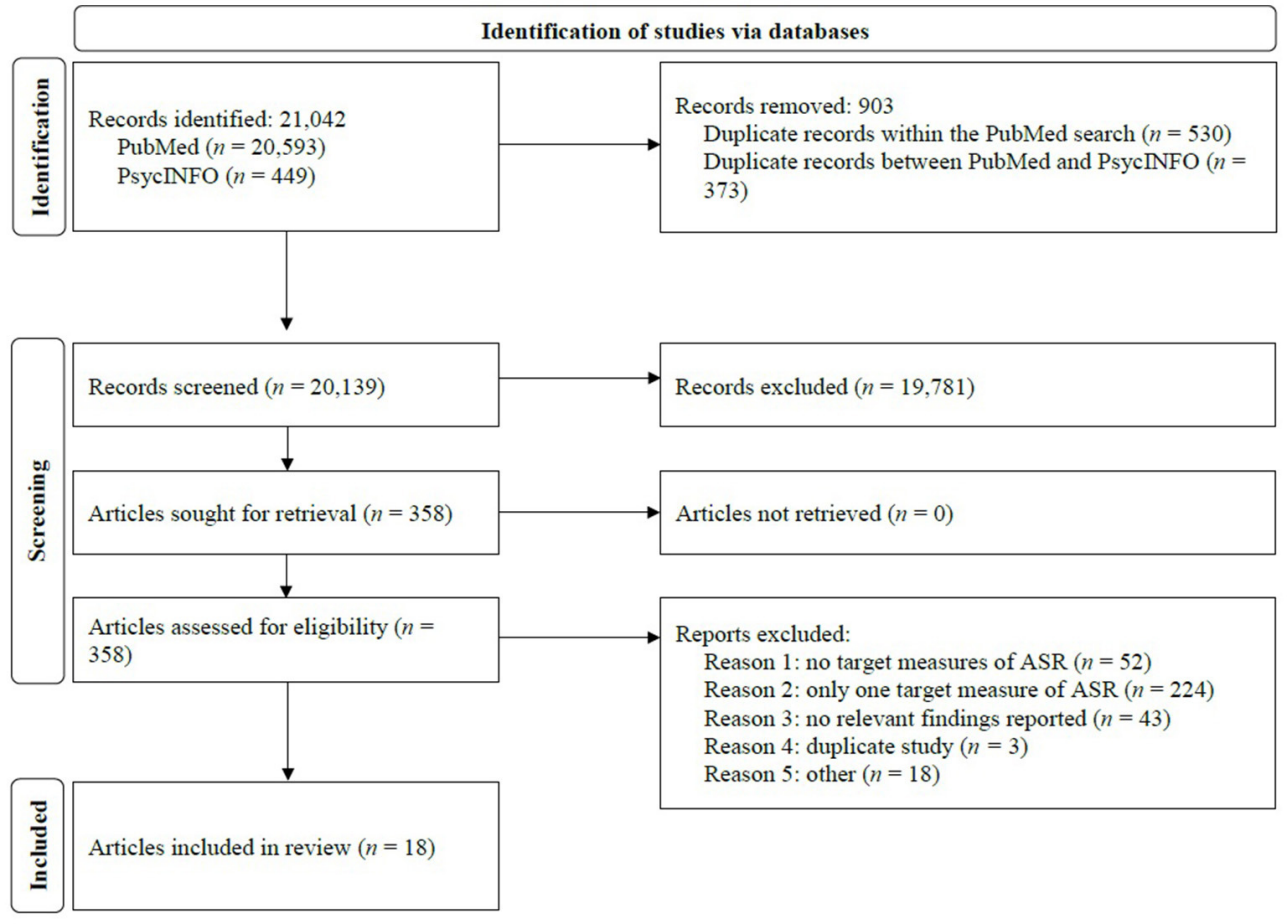
Flow chart of the identification, selection, and inclusion of the retrieved articles.

MAP and NM independently screened titles and abstracts of the articles identified against the study selection criteria after removal of duplicates. Specifically, MAP reviewed all articles from the PsycINFO search. NM reviewed 10,205 articles from the PubMed search, while MP reviewed the rest of the PubMed search articles. The full text of articles appearing to meet eligibility were then individually reviewed and evaluated for final eligibility by NM and MAP. To ensure quality control, approximately 37% of the articles retrieved were double coded and were in high agreement regarding inclusion or exclusion (*k* = 0.83). Any disagreements were resolved through discussion and TGP was consulted in the final selection stage. Eighteen articles met the inclusion criteria and are included in this review. The flow chart of the identification and selection of the reviewed articles is presented in Figure 1.

### Data Extraction

A standardized data extraction form was used to gather the following information: (1) author(s) and publication year, (2) sample size, (3) age, (4) ethnicity/location, (5) assessment tools, (6) implementation, and (7) results/selected findings. This information was extracted to a spreadsheet by NM and checked by MP for accuracy. The results of the review are presented as a narrative summary below and in Table 2.

**Table 2:** Characteristics and selected findings of included studies.

**References**	**Sample size**	**Age**	**Ethnicity/location**	**Assessment tools**	**Implementation**	**Results/selected findings**
**Questionnaire with questionnaire comparisons**						
Koch and Pollatos (33)	Time 1: 1,657 52.1% female 47.9% male Time 2: 1,610 51.9% female 48.1% male	6–11 years *M* = 8.38 *SD* = 0.95	Not provided/ Germany	CEBQ DEBQ	CEBQ: parent report (EOE, FR); collected at T1 & T2 DEBQ: parent report for child (EXE); collected at T1 & T2	Time 1 FR + correlated with Time 1 EXE (*r* = 0.49, *p* < 0.001) Time 2 FR + correlated with Time 2 EXE (*r* = 0.46, *p* < 0.001) Time 2 FR + correlated with Time 1 EXE (*r* = 0.38, *p* < 0.001) Time 2 EXE + correlated with Time 1 FR (*r* = 0.43, *p* < 0.001)
Tan & Holub, (32)	95 46 females 49 males	4–9 years *M* = 6.7 *SD* = 1.2	White = 43% Hispanic = 18% Biracial = 17% Asian = 15% Black = 6% Middle Eastern = 1%	SRES DEBQ	SRES: parent report DEBQ: parent report for child (EME)	SRES—correlated with child EME (*r* = −0.30, *p* < 0.01)
Powell et al. (34)	265	2–7 years *M* = 4.17 *SD* = 1.01	Not provided for children	SRES CEBQ	SRES: parent report CEBQ: parent report (EOE, FR)	SRES—correlated with EOE (*r* = −0.43, *p* < 0.001) SRES—correlated with FR (*r* = −0.45, *p* < 0.001)
**Questionnaire with observation comparisons**						
Cecil et al. (35)	74 37 females 37 males	6–9 years *M* = 92.1 months *SD* = 11.4 months	Not provided/Scotland	CEBQ COMPX	CEBQ: parent report COMPX: school setting, 3 preload conditions of drink & muffin, lunch tray as meal	No significant correlations between CEBQ & deviation scores (% of deviation from perfect compensation); data not shown.
Moens and Braet (36)	52 36 females 16 males	7–13 years *M* = 10.13 *SD* = 1.62	Not provided/Europe	DEBQ EAH task	DEBQ: child self-report (adult version slightly adjusted for children); composite score of EME, EXE EAH: standard procedure; after a dinner meal satiety assessed; followed by 20 min free access to snacks	No significant correlations reported In a logistic regression (controlling for 8 other variables such as child gender, mother and child BMI, SES, & maternal feeding practices), children's report of external and emotional eating (composite score) was positively associated with greater eating in the absence of hunger (*p* < 0.05).
Munsch et al. (37)	41 23 females 18 males all overweight (BMI > 85%ile)	8–12 years females *M* = 9.60 *SD* = 1.5 males *M* = 10.9 *SD* = 1.5	Not provided/Europe	DEBQ-K Preload paradigm	DEBQ-K: German version for children, child self-report; tendency toward overeating score computed by averaging EME & EXE subscales Preload paradigm: atypical procedures, participants received a drink preload or no preload followed by 'taste test' of different flavored “crèmes”; conducted in lab; participants received only one of the preload conditions	“Children with a lower tendency toward overeating decreased their food intake after having received a preload whereas children with a higher tendency toward overeating did not alter their food intake in response to a preload (interaction between preload and tendency toward overeating, *F*_1, 37_ = 3.22, *p* = 0.081).” (p. 101)
Leung et al. (26)	380 190 females 190 males	3–4 years *M* = 4.1 *SD* = 0.54	Non-Hispanic: White = 55.79% Black = 15.53% Biracial/Multiracial = 16.58% Hispanic any race = 11.32%	CEBQ EAH task	CEBQ: parent report (FR, EOE, SR) EAH: after a breakfast meal at school satiety assessed; followed by 10 min free access to snacks	EAH + correlated with FR (*r* = 0.19, *p* < 0.001) EAH + correlated with EOE (*r* = 0.15, *p* < 0.01) No significant correlation between EAH & SR (*r* = 0.01, *p* not provided).
Mallan et al. (38)	37 21 females 16 males	Time 1: *M* = 24.1 months *SD* = 0.7 months Time 2: 3.7-4.5 years	Not provided/ Australia	CEBQ EAH task	CEBQ: parent report (FR, SR, SE) EAH task: conducted at home by mother; meal followed by satiety rating; 15 min play then 15 min free access to snacks	No significant correlations between CEBQ at Time 1 & EAH intake at Time 2 FR + correlated with EAH (*r* = 0.13, *p* = 0.45) SR—correlated with EAH (*r* = −0.02, *p* = 0.90) SE + correlated with EAH (*r* = −0.01, *p* = 0.96)
Hughes et al. (39)	187 89 females 98 males	4–5 years *M* = 57.4 months *SD* = 5.2 months	Hispanic	CEBQ EAH task	CEBQ: parent report (FR, SR) EAH: standard procedures, standard meal, satiety assessment, free access	No significant correlations between: EAH & SR (*r* = 0.00) EAH & FR (*r* = 0.11)
Powell et al. (40)	65 35 females 34 males	2–4 years *M* = 3.54 *SD* = 1.00	Not provided/UK	CEBQ Eating speed	CEBQ: parent report (SE, SR) Eating speed: coded from mealtime observation, mouthfuls per min	SE—correlated with eating speed (*r* = −0.38, *p* < 0.001) SR—correlated with eating speed (*r* = −0.31, *p* < 0.01)
Tan et al. (41)	91 39 females 52 males	Time 1: 26–29 months *M* = 27.33 *SD* = 0.57 Time 2: 33 months	Hispanic non-White = 40.7%	CEBQ EAH task	CEBQ: toddler version, parent report (FR, SR) EAH task: standard procedures; lunch meal at home; followed by 20 min free access to foods	No significant correlations between: Time 1 EAH & Time 1 SR (*r* = −0.07) Time 1 EAH & Time 1 FR (*r* = 0.14) Time 2 EAH & Time 1 SR (*r* = 0.04) Time 2 EAH & Time 1 FR (*r* = 0.06)
Fogel et al. (42)	195 96 females 99 males	Time 1: 4.5 years ± 2 months Time 2: 6 years ± 2 months	Chinese (*n* = 105) Indian (*n* = 38) Malay (*n* = 51) Singapore	CEBQ Eating rate Bite size	CEBQ: parent report (all subscales) Eating rate: observed lunch meal Bite size: observed lunch meal. Energy intake: *ad libitum* lunch buffet meal with parent at Time 1; vegetarian fried rice without parent at Time 2	Time 1: Eating rate & SE—correlated (*r* = −0.14, *p* < 0.05) No significant correlations between: Eating rate & FR (*r* = 0.03, *p* not provided) Eating rate & EOE (*r* = −0.05, *p* not provided) Eating rate & SR (*r* = −0.06, *p* not provided) Bite size & FR (*r* = 0.07, *p* not provided) Bite size & EOE (*r* = −0.03, *p* not provided) Bite size & SR (*r* = −0.08, *p* not provided) Bite size & SE (*r* = −0.02, *p* not provided) Time 2: Eating rate—correlated with SR (*r* = −0.17, *p* < 0.05) Eating rate—correlated with SE (*r* = −0.30, *p* < 0.001) No significant correlations between: Eating rate & FR (*r* = 0.10, *p* not provided) Eating rate & EOE (*r* = 0.01, *p* not provided) Bite size & FR (*r* = −0.04, *p* not provided) Bite size & EOE (*r* = −0.02, *p* not provided) Bite size & SR (*r* = −0.13, *p* < 0.10) Bite size & SE (*r* = −0.01, *p* not provided)
Boone-Heinonen et al. (43)	454 222 females 232 males	2–5 years *M* = 45.2 months *SD* = 9.7 months	Non-Hispanic: White (*n* = 247) Black (*n* = 88) Biracial/Other (*n* = 73) Hispanic any race (*n* = 45)	CEBQ EAH task	CEBQ: parent report (FR, SR) EAH task: cohort 1: standard procedures; after breakfast satiety assessed; followed by 10 min free access to snack; cohort 2: after lunch 10 min free access to snacks	EAH + correlated with FR (*r* = 0.18, *p* not provided) No significant correlation between EAH & SR (*r* = −0.04)
Blissett et al. (24)	62 29 females 33 males	3–5 years *M* = 46.0 months *SD* = 6.8 months	British White = 89%	CEBQ EAH task	CEBQ: parent report (FR, EOE, SR) EAH task: conducted after mood induction task; ~30 min between meal & EAH; 4 min free access	No significant correlation between: EAH kcals & FR (*r* = 0.00, *p* not provided, *n* = 29) EAH kcals & EOE (*r* = 0.10, *p* not provided, *n* = 21) EAH kcals & SR (*r* = −0.23, *p* not provided, *n* = 30)
**Observation with observation comparisons**						
Orlet Fisher et al. (44)	35 18 females 17 males	2.9–5.1 years *M* = 4.0 *SD* = 0.5	Black (*n* = 1) Asian (*n* = 4) Non-Hispanic White (*n* = 28) Hispanic (*n* = 2)	EAH task Bite size	EAH task: standard procedure; after one of the lunches hunger assessed; followed by small taste test of snack foods and 10 min of free access to snacks Bite Size: average bite size; total grams divided by total # of bites taken	No significant correlation between EAH & bite size (*r* = 0.20, *p* = not provided, *n* = 23).
Remy et al. (27)	236 109 females 127 males	3–6 years *M* = 4.5 *SD* = 0.06	not provided/France	EAH task COMPX	EAH: standard procedure COMPX: standard procedure	No significant correlation between EAH & COMPX (*r* = 0.05; *p* = 0.46)
**Questionnaire with questionnaire and observation with questionnaire comparisons**						
Madowitz et al. (45)	117 62 females 55 males all overweight (BMI >85%tile)	8–12 years *M* = 10.42 *SD* = 1.35	White = 54% Black = 14% Multi-Race = 20% Other = 12%	EAH task EAH-C EAH-PC	EAH task: standard procedure; after dinner meal, satiety, hunger, & fullness assessed; followed by small taste test of snack foods & 10 min free access to snacks; EAH% calculated to get % of daily caloric needs eaten during EAH task EAH-C: questionnaire, child self-report EAH-PC: questionnaire, parent report for child.	EAH-C total score + correlated with EAH-PC total score (*r* = 0.34, *p* < 0.001) EAH-C total score + correlated with EAH-PC EXE (*r* = 0.25, *p* < 0.01) No significant correlation between: EAH% & EAH-C total score (*r* = −0.04, *p* not provided) EAH% & EAH-PC total score (*r* = −0.12, *p* not provided) EAH% & EAH-C EXE (*r* = 0.01, *p* not provided) EAH% & EAH-PC EXE (*r* = −0.08, *p* not provided) EAH-C EXE & EAH-PC total score (*r* = 0.17, *p* not provided) EAH-C EXE & EAH-PC EXE (*r* = 0.18, *p* not provided)
**Observation with questionnaire and observation with observation comparisons**						
Carnell and Wardle (25)	111 55 females 56 males	4–5 years	British White = 74%	CEBQ Eating rate COMPX EWH task	CEBQ: parent report (FR, SR) Eating rate: average across meals COMPX: used disguised and undisguised preloads EWH task: modified EAH by offering only 1 food during free access	No significant correlations between: Average eating rate & EWH intake (*r* = 0.13, *p* < 0.10, *n* = 100) Average eating rate & COMPX undisguised (*r* = −0.23, *p* < 0.10, *n* = 68) Average eating rate & COMPX disguised (*r* = −0.17, *n* = 91) COMPX disguised & EWH (*r* = −0.06, *n* = 86) COMPX undisguised & EWH (*r* = −0.12, *n* = 61) COMPX undisguised & COMPX disguised (*r* = 0.17, *n* = 57) Simple linear regressions: SR + associated with EWH intake (*r*^2^ = 0.11, *p* = 0.001, *n* = 98) SR + associated with average eating rate (*r*^2^ = 0.11, *p* = 0.001, *n* = 101) FR + associated with average eating rate (*r*^2^ = 0.06, *p* < 0.009, *n* = 109) No significant correlations between: SR with COMPX disguised (*r*^2^ = 0.003, *p* = 0.471, *n* = 89) SR with COMPX undisguised (*r*^2^ = 0.05, *p* = 0.072, *n* = 66) FR with EWH intake (*r*^2^ = 0.006 *p* < 0.45, *n* = 98) FR with COMPX disguised (*r*^2^ = 0.02, *p* < 0.21, *n* = 89) FR with COMPX undisguised (*r*^2^ = 0.001, *p* < 0.86, *n* = 66)

*Definitions of abbreviations as they appear by column: M, mean; SD, standard deviation; BMI, Body Mass Index; CEBQ, Children's Eating Behaviors Questionnaire; DEBQ, Dutch Eating Behavior Questionnaire; SRES, Self-Regulation in Eating Scale; COMPX, % of energy compensation; EAH, Eating in the Absence of Hunger; DEBQ-K, Dutch Eating Behavior Questionnaire-Kinder; EWH, Eating Without Hunger; EAH-C, Eating in the Absence of Hunger-Child self-report; EAH-PC, Eating in the Absence of Hunger-Parent report of child; EOE, emotional overeating; FR, food responsiveness; EXE, external eating; EME, emotional eating; SR, satiety responsiveness; SE, slowness in eating*.

## Results

Eighteen studies were eligible for inclusion, representing studies measuring common ASR constructs as assessed by: (1) four unique self-report questionnaires, three of which included multiple versions: CEBQ (two versions: CEBQ and CEBQ for toddlers), DEBQ (three versions: parent-report for child behaviors, modified parent version for child self-report, German version for child self-report), SRES, EAH (parent-report for child behaviors and child self-report); and (2) four unique observational tasks, two of which were implemented slightly differently across studies: compensation trials/preload paradigm, EAH/Eating Without Hunger (EWH) tasks, eating rate/speed, and bite size. Implementation information and deviations from typical procedures are presented in Table 2.

### Characteristics of the Included Studies

Table 2 also presents study characteristics and selected findings of the included articles [i.e., author(s) and publication year, sample size, age, ethnicity/location, assessment tools, assessment implementation, and results/selected findings]. Most of the included studies were conducted outside the United States (*n* = 10), with eight conducted in Europe, one in Australia, and one in Singapore. Seven were conducted within the United States and one did not report location of their subjects. Of the studies conducted outside of the United States, only three reported information on race/ethnicity. Of the studies conducted within the US, most included participants from differing ethnic/racial backgrounds, although the majority was comprised of predominantly White participants. Gender distribution was approximately equal throughout all studies and child ages ranged from 2 to 13 years.

### Methodologies Used to Assess ASR Constructs

Of the 18 studies that met eligibility, three studies compared data between different self-report questionnaires: Koch and colleagues (33) compared the CEBQ and the DEBQ; Tan and Holub (32) compared the SRES and the DEBQ; and Powell and colleagues (34) compared the SRES and the CEBQ. Regarding comparisons of questionnaires with observational tasks, nine studies compared the CEBQ with various tasks: compensation trials (35); eating rate/speed (40, 42) and bite size (42); or EAH (24, 26, 38, 39, 41, 43). One study compared the DEBQ (child self-report) with the EAH task (36) and another compared the DEBQ-K (child self-report; German version) with a preload paradigm (37). Three studies compared data between different observational tasks: the EAH was compared to bite size (44) and compensation trials (27); Carnell and Wardle (25) compared the following tasks to each other, compensation trials (disguised and undisguised), eating rate, and EWH. This latter study also compared all observational tasks to the CEBQ. Finally, one study conducted by Madowitz and colleagues (45), using the EAH task and EAH questionnaires (parent- and child-report), compared both versions of the EAH questionnaire to the task as well as to one another.

### Inter-relatedness of ASR Constructs

Overall, the majority of significant associations were seen in cohort studies involving multiple self-report questionnaires of ASR. Specifically, in the Koch and colleagues (33) study, food responsiveness (CEBQ) was positively associated with external eating (DEBQ) at the 1st time point of the study (*r* = 0.49, *p* < 0.001) and remained significant at the 2nd time point (*r* = 0.46, *p* <0.001). Additionally, significant positive associations were found for these constructs across time points with Time 2 food responsiveness correlating positively with external eating at Time 1 (*r* = 0.38, *p* <0.001) and Time 2 external eating positively correlating with Time 1 food responsiveness (*r* = 0.43, *p* <0.001). Powell and colleagues (34) also found strong associations between subscales of two questionnaires. Child eating self-regulation (SRES) was negatively associated with both emotional overeating (*r* = −0.43, *p* <0.001) and food responsiveness (*r* = −0.45, *p* <0.001) from the CEBQ. The SRES was also negatively associated with emotional eating from the DEBQ (*r* = −0.30, *p* <0.01) in the Tan and Holub (32) study, but the association was moderate.

Most of the 11 studies comparing self-report questionnaires to observational tasks showed either no significant associations (4 studies) (24, 35, 39, 41) or weak and no associations (3 studies) (26, 38, 43). Two of the 11 studies showed moderate associations only between eating rate/speed and CEBQ subscales: negative association with slowness in eating (*r* = −0.30, *p* <0.001) (42); and negative association with both slowness in eating and satiety responsiveness (*r* = −0.38, *p* <0.001, *r* = −0.31, *p* <0.01, respectively) (40). Between the two studies comparing observational tasks to other tasks, none showed significant associations (27, 44).

Carnell and Wardle (25) and Madowitz et al. (45) found some significant moderate associations in their studies that used mixed methods and more than one ASR observational task or self-report questionnaire. Carnell and Wardle (25) showed that satiety responsiveness was positively associated with both EWH and average eating rate (*r* = 0.33, *p* = 0.001, *r* = 0.33, *p* = 0.01, respectively), and that food responsiveness was also positively associated with average eating rate (*r* = 0.25, *p* = 0.009). However, no significant associations were shown between observational tasks. On the other hand, Madowitz and colleagues (45) found significant associations only between the parent- and child-report versions of the same questionnaire (EAH). The total scores of the child self-report were moderately associated with those of the parent-report of child behaviors (*r* = 0.34, *p* <0.001), and weakly to moderately associated to the external eating subscale of the parent-report (*r* = 0.25, *p* <0.01).

Within the studies comparing ASR questionnaires (32–34, 45), only Madowitz et al. (45) used different raters (i.e., parent and child) for children's behaviors with moderate and weak to moderate associations (*r* = 0.34, *p* <0.001, *r* = 0.25, *p* <0.01, respectively). The strength of these associations is lower than those found in the other three studies that compared data from the same rater (i.e., parent-report). Moreover, the evidence of association strength in the Madowitz and colleagues (45) study matches the strength of association strength (i.e., moderate) in three studies that compared data from self-report questionnaires and observational tasks (25, 40, 42). On the other hand, the moderate associations were found in these three studies, while the majority of the studies comparing self-report questionnaire with observational task data showed either weak or no significant associations. In contrast, within studies comparing data from several self-report questionnaires, all four studies showed significant associations.

### ASR and Child BMIz

Of the 18 studies included in this review, 11 examined associations between at least one measure of ASR constructs and child BMIz or an equivalent score. Most studies used standard methods for calculating child BMIz (e.g., CDC standards) except three studies: weight-for-length z score (WLZ) (41) and similar procedures (35, 37). Henceforth, BMIz will be used to describe child weight status scores. Of the 11 studies, four used self-report questionnaires to measure ASR (32, 33, 36, 37), three used observational tasks (27, 35, 44), and four used both questionnaires and tasks (24, 38, 39, 41).

Among the four studies examining associations between self-report questionnaires and child BMIz, only two studies found associations (32, 33). Emotional overeating and food responsiveness (CEBQ) were positively associated with BMIz (*r* = 0.17, *p* <0.001 and *r* = 0.45, *p* <0.001, respectively) (33); external eating (DEBQ) was also positively associated with BMIz (*r* = 0.21, *p* <0.001) (33). It should be noted that these associations were found in a larger sample [*n* = 1,657 (33)]. Self-regulation in eating (SRES) was negatively associated with BMIz (*r* = −0.30, *p* <0.01) (32). The other two studies which measured external and emotional eating (DEBQ) found no associations (36, 37).

Among the three studies examining associations between observational tasks and child BMIz, only one study found an association—bite size was positively associated with BMIz (*r* = 0.55, *p* <0.01) (44). No associations were found between the EAH task (27) or the compensation trials (27, 35) with BMIz.

All four studies that examined associations between both self-report questionnaires and observational tasks with child BMIz, used the CEBQ and the EAH task to measure ASR (24, 38, 39, 41). Of the four studies, three found associations: a negative association with satiety responsiveness [*r* = −0.42, *p* = 0.015 (38); *r* = −0.24, *p* <0.01 (39); *r* = −0.28, *p* <0.01 (41)] was found in all three studies; a positive association with food responsiveness (*r* = 0.15, *p* <0.05) (39) was found in one study. The EAH task was associated with BMIz in one of the four studies (*r* = 0.20, *p* <0.01) (39).

## Discussion

This narrative review was aimed at examining associations between of common child ASR constructs as assessed by at least two methods/measures. The aim was to examine these constructs both within and across self-report questionnaires and/or observational tasks. A total of 18 studies met eligibility criteria and were included in the review. The three studies comparing constructs using self-report questionnaires showed the most correspondence between different ASR constructs. In contrast, the two studies comparing ASR constructs using different observational tasks showed no correspondence. Furthermore, among the 11 studies comparing self-report questionnaires to observational tasks, two studies showed moderate correspondence and nine studies showed weak and/or no associations. As mentioned previously, the remaining two studies compared constructs within and across methodologies and showed weak and/or no associations.

Among the three studies using self-report questionnaires, three questionnaires were used to measure correspondence between constructs—emotional overeating (CEBQ) positively associated with external eating (DEBQ) (33); self-regulation of eating (SRES) negatively associated with emotional eating (DEBQ) (32); and self-regulation of eating (SRES) negatively associated with emotional overeating and food responsiveness (CEBQ) (34). That emotional overeating and external eating were positively associated could be explained by the shared elements of eating without regard to hunger and satiety cues. This correspondence is in line with the construct definitions provided in Table 1. Similarly, the negative association between self-regulation of eating as measured by the SRES and emotional overeating/emotional eating may reflect that responsiveness to internal cues of hunger is diminished by emotional overeating. The negative association found between SRES and food responsiveness could reflect the idea that response to external cues (e.g., sight and smell) and the response to internal cues represent opposite ends of a continuum.

However, the correspondence between these constructs, as measured by self-report questionnaires, could partly be due to method biases that can result when the data is provided by the same source/rater or by the measurement context in which the data was obtained. Apart from the Madowitz and colleagues (45) study, the studies reporting on associations within questionnaires gathered data from the same rater (32–34). When the same source provides data, an “artifactual covariance” can be created between the variables in an effort to create a consistent “story” (or *consistency motif*) between the rater's cognitions and responses (46). Additionally, the use of the same rater can generate an *implicit theory* which may “affect attention to and encoding of ratee behaviors as well as later recall” (p. 599) (47). For example, a parent completing questionnaires on their child's eating behaviors may bias their responses based on an overall view they have of their child, which may not necessarily be specific to eating. If a child is difficult, the parent may be biased to create a consistent “story” of their child's eating as being difficult. The measurement context in which the raters provide responses can also be a source of bias. For example, the current mood state of the rater as well as the time of day and location of assessment may impact responses (46). Specifically, a rater's retrieval of information may affect questionnaire completion because of the presence of “common contextual cues” influencing their memory and thus, associations between variables (46).

Among the two studies comparing constructs using only observational tasks (27, 44) and one study that examined constructs within and across methods (25), four assessments tools were used including the eating in the absence of hunger task, various types of compensation trials, and the measurements of eating rate and bite size. Across these different observational tasks, none of the constructs showed correspondence. One reason for the lack of correspondence across observations could stem from the nature of observations—the capture or snapshot of behavior at a single point in time. It is possible that observed data capture state-based behaviors, whereas self-report questionnaire data capture behaviors that parents observe or children engage in across multiple occasions and over an extended period of time—trait-based behaviors.

Among the 11 studies comparing self-report questionnaires and observational tasks of ASR, assessment tools included observations of eating rate, bite size, EAH, and compensation trials as well as the CEBQ (i.e., satiety responsiveness, food responsiveness, slowness in eating, and emotional overeating), and the DEBQ (i.e., emotional and external eating). Two additional studies examining constructs within and across methods also used most of these measures as well as the EAH questionnaire. Of these 13 studies, the most common association found was between eating rate and the CEBQ subscales of satiety responsiveness, food responsiveness, and slowness in eating (25, 40, 42). This common finding may be explained by the simplicity of the eating rate observations. Measuring eating rate can be considered fairly simple, direct, and practical compared to other observational assessments involving multiple steps over a longer period of time. In addition to the eating rate finding, mixed results (weak or no associations) were found across seven studies comparing EAH and the CEBQ subscales (24–26, 38, 39, 41, 43), while no associations were found between compensation trials (25, 35), bite size (44) and the CEBQ subscales, and between the DEBQ and a preload paradigm [a modified form of the compensation trials] (37). Interestingly, neither the parent- nor the child-report of the EAH questionnaire were associated with the EAH task.

Compensation trials did not reveal any significant associations with any ASR measures. One possible reason for this lack of findings is that the percent of compensation shown by children in these tasks usually shows a wide range of values and it is not clear how much of this variation (based on only a single pair of meals) represents stable individual differences in children's ASR vs. variability due to the many situational factors that can affect children's consumption on a single pair of occasions (time of day, child hunger, child mood, child food preferences, etc.). As part of an evaluation of a childhood obesity prevention program, Hughes and colleagues (48) found that although the COMPX scores (i.e., % of energy compensation) showed the expected relationships with child weight status, there was no significant stability in this variable over a 9- to 10-week period in either their prevention or control groups. This suggests that although this variable may be useful in the comparison of groups of children, a single pair of meals may not be sufficient to yield stable measure of individual differences in ASR. Additionally, the lack of findings with the EAH task, may stem from socialization influences that could be affecting children's behaviors during this task. Hughes and colleagues (48) have suggested that tasks, such as compensation trials and EAH, may not be effective measures of ASR with certain samples (i.e., Hispanic children from low-income backgrounds) for various reasons. For example, it is highly likely that these children experience high food insecurity at home, or the foods provided during the tasks are unfamiliar or not culturally congruent to the children. Moreover, children show wide variability in their responses to the EAH task, and individual differences may reflect both situational factors as well as individual differences in ASR. In the Hughes and colleagues (48) study, however, significant stability was shown over a 9- to 10-week period in both the prevention (*r* = 0.50) and control (*r* = 0.32) groups.

The lack of associations between ASR constructs as measured by self-report questionnaires and observational tasks has been shown in studies of adults as well (49). Interestingly, similar to the findings from this narrative review, Creswell et al. (49) found that associations between self-report questionnaires and observed computerized tasks were either weak or non-significant. Additionally, the self-report questionnaires showed associations with outcomes, whereas the computerized tasks showed weak or no associations with outcomes. This is in line with findings from the current review showing significant associations between ASR constructs and child weight outcomes across five studies using self-report questionnaires. Specifically, these studies showed significant associations between ASR constructs and child BMIz across five studies using self-report questionnaires. Specifically, satiety responsiveness, food responsiveness, and emotional overeating from the CEBQ (33, 38, 39, 41), external eating from the DEBQ (33), and child self-regulation in eating (32) were associated with child BMIz. In contrast, only two studies showed significant associations with child BMIz using observational tasks (39, 44). The findings from this review are consistent with a recent systematic review of the CEBQ subscales and child weight (50). Among studies comparing observational tasks and child BMIz, only two constructs showed associations—bite size (44) and EAH (39). Interestingly, EAH was associated with child BMIz in only one (39) of five studies (24, 27, 41, 44), despite the fact that EAH has consistently been shown to be associated with child weight status (51). These associations were specific to studies that involved more than one ASR measure and constitutes a small subset of studies looking at associations of ASR measures with weight status. The association found by Hughes et al. (39) is consistent with previous reports among these constructs in young children (51).

Findings from this narrative review should be considered in light of its limitations. Inclusion in his review required that each study assessed at least two ASR measures and reported associations. Furthermore, although many factors impact ASR in children, including biopsychological (e.g., genes, hormones, executive functioning) and family and community processes (7, 21), the current review focused on the intrapersonal factors of common ASR constructs. Moreover, only a subset of published articles (i.e., the 18 included in this review) reported associations between the measured ASR constructs with over 40 identified that did not present associations. This limits the interpretation of the findings, because if more data were available, the relationship between the targeted measures may have presented differently.

### Future Research and Implications

It is thought that questionnaire-based measures have clear advantages over observational tasks for a number of reasons. Specifically, questionnaires (1) involve little participant burden for young children as parents often report on child behaviors, (2) present relatively low participant burden for parents, and (3) are more feasible to administer compared to many observational protocols that involve multiple steps administered by trained research staff. In this sense, questionnaires have obvious advantages for measurement in large epidemiological studies and interventions as well as for rapid identification of at-risk children in healthcare settings.

Future research is needed to more clearly define the various ASR constructs, their expected correspondence, as well as the strength of that correspondence. Additionally, as other scholars have suggested, current literature would benefit from studies considering the biology of the child as well as the child's immediate and more distal environments (7). The use of mixed methods comprised of existing tools, as well as conducting the same assessments over a shorter period of time (e.g., across 10 days) will better determine whether these constructs measure a state vs. a trait. Longitudinal research will provide evidence of predictability. Taken together, this additional information and identifying which ASR constructs are most effective can inform efforts toward successful childhood obesity programs that promote healthful eating behaviors in families. Further, investigating the relations between ASR constructs and child weight, among youth with and without overweight/obesity and their parents, fosters a better understanding for predicting obesity risk in children.

## Author Contributions

MP, SH, TP, and JF contributed to conception and design of the review. MP and NM conducted the article search and review and organized the database. TP approved the final list of articles. MP wrote the first draft of the manuscript. SH, TP, and JF wrote sections of the manuscript. All authors contributed to the revision of the manuscript and read and approved the submitted version.

## Funding

This work is a publication of the United States Department of Agriculture (USDA/ARS) Children's Nutrition Research Center, Department of Pediatrics, Baylor College of Medicine (Houston, TX) funded in part by the USDA/ARS (Cooperative Agreement 58-3092-0-001).

## Author Disclaimer

The contents of this publication do not necessarily reflect the views or policies of the USDA, nor does mention of trade names, commercial products, or organizations imply endorsement from the US government.

## Conflict of Interest

The authors declare that the research was conducted in the absence of any commercial or financial relationships that could be construed as a potential conflict of interest.

## Publisher's Note

All claims expressed in this article are solely those of the authors and do not necessarily represent those of their affiliated organizations, or those of the publisher, the editors and the reviewers. Any product that may be evaluated in this article, or claim that may be made by its manufacturer, is not guaranteed or endorsed by the publisher.
